# The Influence of an Acute Administration of Cannabidiol or Rivastigmine, Alone and in Combination, on Scopolamine-Provoked Memory Impairment in the Passive Avoidance Test in Mice

**DOI:** 10.3390/ph17060809

**Published:** 2024-06-20

**Authors:** Marta Kruk-Slomka, Tomasz Slomka, Grazyna Biala

**Affiliations:** 1 Department of Pharmacology and Pharmacodynamics, Medical University of Lublin, 4a Chodzki Street, 20-093 Lublin, Poland; grazyna.biala@umlub.pl; 2 Department of Information Technology and Medical Statistics with e-Health Laboratory, Medical University of Lublin, Jaczewskiego 4 Street, 20-954 Lublin, Poland; tomasz.slomka@umlub.pl

**Keywords:** cannabidiol, rivastigmine, scopolamine-induced amnesia, passive avoidance, mice

## Abstract

Memory is one of the most important abilities of our brain. The process of memory and learning is necessary for the proper existence of humans in the surrounding environment. However, sometimes there are unfavourable changes in the functioning of the brain and memory deficits occur, which may be associated with various diseases. Disturbances in the cholinergic system lead to abnormalities in memory functioning and are an essential part of clinical symptoms of many neurodegenerative diseases. However, their treatment is difficult and still unsatisfactory; thus, it is necessary to search for new drugs and their targets, being an alternative method of mono- or polypharmacotherapy. One of the possible strategies for the modulation of memory-related cognitive disorders is connected with the endocannabinoid system (ECS). The aim of the present study was to determine for the first time the effect of administration of natural cannabinoid compound (cannabidiol, CBD) and rivastigmine alone and in combination on the memory disorders connected with cholinergic dysfunctions in mice, provoked by using an antagonist of muscarinic cholinergic receptor—scopolamine. To assess and understand the memory-related effects in animals, we used the passive avoidance (PA) test, commonly used to examine the different stages of memory. An acute administration of CBD (1 mg/kg) or rivastigmine (0.5 mg/kg) significantly affected changes in scopolamine-induced disturbances in three different memory stages (acquisition, consolidation, and retrieval). Interestingly, co-administration of CBD (1 mg/kg) and rivastigmine (0.5 mg/kg) also attenuated memory impairment provoked by scopolamine (1 mg/kg) injection in the PA test in mice, but at a much greater extent than administered alone. The combination therapy of these two compounds, CBD and rivastigmine, appears to be more beneficial than substances administered alone in reducing scopolamine-induced cognitive impairment. This polytherapy seems to be favourable in the pharmacotherapy of various cognitive disorders, especially those in which cholinergic pathways are implicated.

## 1. Introduction

Memory disorders are typical symptoms from many neurodegenerative disorders, including Alzheimer’s disease (AD), which is the most common and most serious disease of this type. AD is a progressive process and causes gradual, irreversible damage to the brain structures due to the deposition of two pathological structures: amyloid plaques (β-amyloid) and tau protein, causing the death of neurons. Based on the results of this pathology, as well as other diseases involving memory disorders, degeneration of cholinergic neurons occurs very often, the level of Ach—a key neurotransmitter for memory regulation—decreases, and impairment of cholinergic transmission follows. In connection with these, the most important symptom of AD is memory loss and cognition-related disturbances [1,2]. Currently, the treatment of cognitive disorders is based only on symptomatic management. The decrease in the level of ACh is still the main target of this kind of treatment. Based on that, inhibitors of acetylcholinesterase (AChEI), i.e., an enzyme that breaks down Ach, are the most popular group of drugs already registered and applied in the USA and/or in Europe to control/treat memory disorders connected with AD. Various drugs from AChEI (e.g., donepezil, galantamine, rivastigmine) enhance cholinergic function, by increasing the concentration of ACh through reversible inhibition of its hydrolysis by cholinesterase. These drugs can cause more or less therapeutic effects measured by cognitive improvement that depends on their level of inhibition of the cholinesterase. However, AChEIs have only a minor effect on the improvement of the patient’s condition, have no influence on other AD-related symptoms, i.e., depression, psychosis, and anxiety, often appearing as a consequence of memory deficits, and can cause several severe adverse effects like nausea, vomiting, diarrhoea, and weight loss [3,4,5].

Due to the lack of the expected effect from the drugs currently used, researchers are still trying to discover the cause of AD development, as it is not fully understood at present. It is important to look for new therapeutic solutions that will eliminate the cause, inhibit the development of the disease, or reverse pathological processes and thus make pharmacotherapy more effective. Additionally, it is unlikely that any drug acting on a single pathway or target will mitigate the complex pathology cascade leading to cognitive disturbances. Therefore, a multifunctional drug approach targeting a number of AD pathologies simultaneously will provide better, wider-ranging benefits than current therapeutic approaches [1,6,7,8].

One of the new possible and very promising strategies for pharmacological modulation/control of memory-related problems connected with cholinergic pathways is associated with the endocannabinoid system (ECS) that include cannabinoid receptors (CB), endocannabinoids (anandamide (AEA) and 2-arachidonoylglycerol (2-AG), and enzymes (fatty acid amide hydrolase (FAAH) and monoglyceride lipase (MAGL) that control the metabolism and degradation of endocannabinoids [8]. Cannabinoid compounds act on two types of CB receptors: CB1, located mainly in the central nervous system (CNS), and CB2, located primarily in the immune system. Thus, cannabis compounds due to a multidirectional mechanism of action are associated with regulating a variety of processes related to memory and learning and may turn out to be a new strategy in the treatment of cognitive syndromes. The most well-known phytocannabinoids, occurring in Cannabis sativa, are tetrahydrocannabinol (THC) and cannabidiol (CBD). Currently, research is being conducted on their effectiveness and safety of use in AD. On the one hand, THC has been shown to reduce AChE activity and prevent the accumulation of β-amyloid, but it is a very strong psychoactive substance. Due to the psychostimulating properties that occur after THC, CBD without psychotropic effects raises more hopes for therapeutic use [9,10]. In animal studies, CBD has been proven to reverse cognitive decline and prevent its further progress, and to enhance neuroplasticity in the brain. In addition, CBD has procognitive, neuroprotective, and anti-inflammatory properties, and inhibits the excessive phosphorylation of the tau protein, as well as protects against Aβ-mediated neurotoxicity and microglial-activated neurotoxicity [11,12,13,14], which may suggest its effectiveness in treating the causes of AD.

Taking into account the dysfunction of cholinergic pathways associated with the pathology of AD and the probable possibility of using cannabinoid compounds in the treatment of this disease, it seems very important to explore the interactions between ECS and cholinergic systems, in the context of more effective pharmacotherapy of cognitive disorders. Perhaps, the hypothesis is that a combination of cholinergic and ECS intervention could potentially provide a more effective therapy for AD. The aim of this study was to assess for the first time the effect of one of the natural cannabinoid compounds, CBD, and one of the commonly used AChEIs, rivastigmine, on memory disorders connected with cholinergic dysfunctions in mice. We examined the impact of acute injections of CBD and rivastigmine administered alone, as well as a combined injection, on the memory acquisition, consolidation, and retrieval impairment provoked by an acute injection of a cholinergic antagonist—scopolamine. Scopolamine is known as a pharmacological model of memory disorders because, by blocking muscarinic cholinergic receptors, it impairs cholinergic transmission and causes disturbances in the formation of cognitive pathways. To assess memory-related processes in mice, we used the passive avoidance test (PA), which allows evaluating different stages of memory depending on the drug treatment.

The obtained results will allow us to broaden the knowledge concerning the role of CBD in all stages of memory: acquisition, consolidation, and restoration, as well as to obtain for the first time results pointing out the effectiveness of the combined use of CBD with the drug used commonly in the pharmacotherapy of AD, e.g., rivastigmine. Such co-administration of CBD and rivastigmine could be more beneficial in reducing scopolamine-induced cognitive impairment in mice than these substances administered alone. Thus, polytherapy of two compounds with different mechanism of action seems to be more effective and safer in humans. For example, CBD and rivastigmine combined therapy could be a potential disease-modifying therapeutic approach with limited adverse effects. Perhaps, the obtained results may contribute to the planning of further studies at the clinical level, in order to search for new combined pharmacological strategies in the treatment of cognitive disorders, especially those in which cholinergic pathways are implicated, e.g., AD.

## 2. Results

In the first step of the experiments, we evaluated the influence of an acute administration of all used compounds: phytocannabinoid compound—CBD, AChEI—rivastigmine, or cholinergic muscarinic receptor antagonist—scopolamine, on the different stages of long-term memory: acquisition, consolidation, and retrieval.

### 2.1. The Influence of an Acute Injection of CBD, Rivastigmine, or Scopolamine on Long-Term Memory in Mice in the PA Test

#### Acquisition, Consolidation, and Retrieval of Memory

One-way ANOVA revealed that the administration of acute ip doses of CBD (1, 5, 30 mg/kg), rivastigmine (0.5, 1, 2.5 mg/kg), or scopolamine (0.1, 0.3, 1 mg/kg) had a statistically significant effect on LI values for:

- Long-term memory acquisition [F(9.87) = 24.21; *p* < 0.0001];
- Long-term memory consolidation [F(9.80) = 16.01; *p* < 0.0001];
- Long-term memory retrieval [F(9.82) = 16.00; *p* < 0.0001].

Additionally, the post hoc Tukey’s test confirmed that CBD only at the highest dose (30 mg/kg) and rivastigmine (at doses of 1 and 2.5 mg/kg) significantly increased LI values in mice compared to those in the vehicle-treated control group:

- Acquisition for memory—(*p* < 0.01 for CBD (30 mg/kg); *p* < 0.001 for rivastigmine (1 and 2.5 mg/kg));
- Consolidation for memory—(*p* < 0.01 for CBD (30 mg/kg); *p* < 0.01 for rivastigmine (1 mg/kg); *p* < 0.001 for rivastigmine (2.5 mg/kg));
- Retrieval for memory—(*p* < 0.001 for CBD (30 mg/kg); *p* < 0.05 for rivastigmine (1 mg/kg); (*p* < 0.001 for rivastigmine (2.5 mg/kg)).

**Figure 1:**
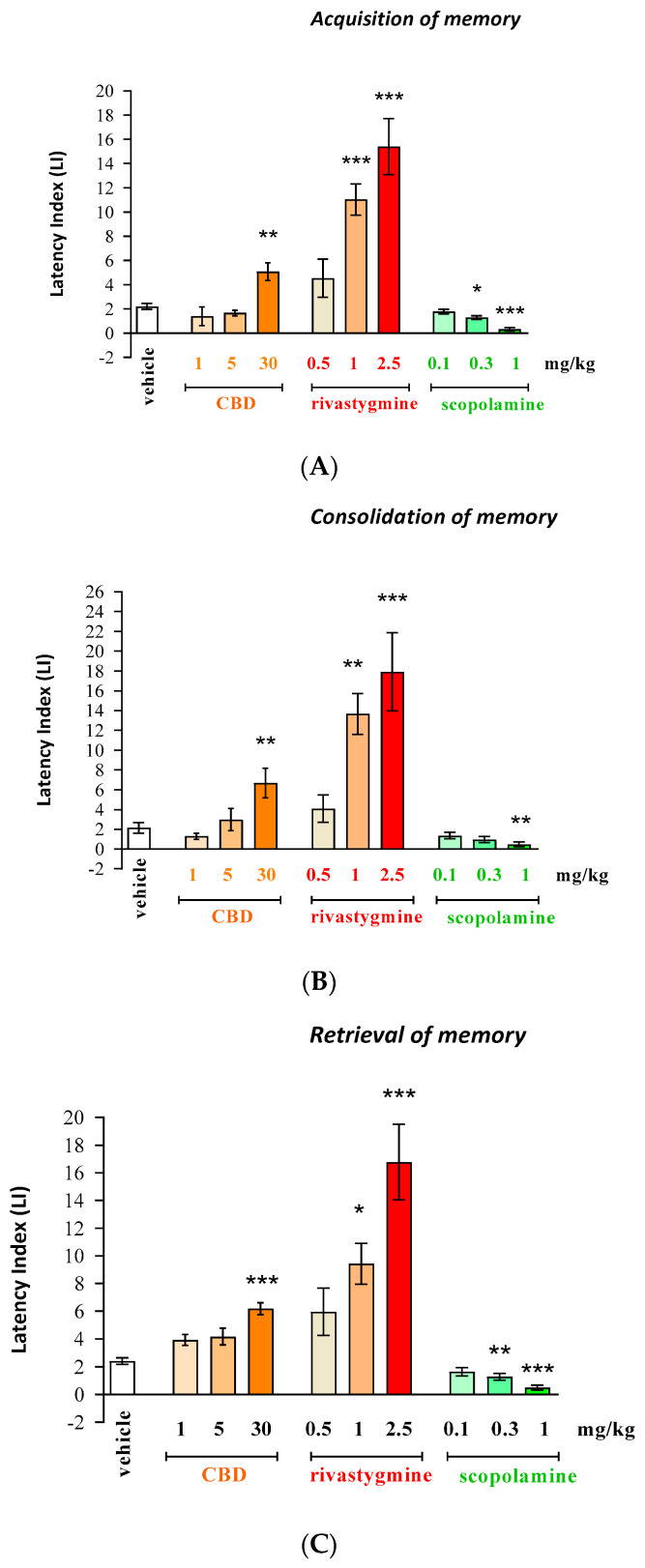
Influence of an acute administration of selected compounds: phytocannabinoid compounds—cannabidiol (CBD); AChEI—rivastigmine or cholinergic muscarinic receptor antagonist—scopolamine on the cognition-related responses expressed as a latency index (LI) during the acquisition (**A**), consolidation (**B**), and retrieval (**C**) of memory using the PA test in mice. CBD (1, 5, 30 mg/kg), rivastigmine (0.5, 1, 2.5 mg/kg), scopolamine (0.1, 0.3, 1 mg/kg), or vehicle were administered 30 min before the first trial (acquisition of memory), immediately after the first trial (consolidation of memory), or before the second trial (retrieval of memory). The second trial was conducted 24 h later; the means ± SEM; * *p* < 0.05; ** *p*< 0.01; *** *p* < 0.001 vs. vehicle-treated group; Tukey’s test; n = 7–12 (the exact number of mice in a test are presented in the table below (Table 1A–C)).

These results indicate that CBD and rivastigmine, at these used doses, improved the long-term acquisition, consolidation, and retrieval of memory processes in the PA test in mice (Figure 1A–C, appropriately).

Moreover, the post hoc Tukey’s test confirmed that an acute injection of scopolamine (at doses of 0.3 and/or 1 mg/kg) significantly decreased LI values in mice compared to the vehicle-treated control group:

- Acquisition of memory—(*p* < 0.05 for dose of 0.3 mg/kg and *p* < 0.001 for dose of 1 mg/kg);
- Consolidation of memory—(*p* < 0.01 for dose of 1 mg/kg);
- Retrieval of memory—(*p* < 0.01 for dose of 0.3 mg/kg and *p* < 0.001 for dose of 1 mg/kg).

These results indicate that scopolamine, at these used doses, impaired the long-term acquisition, consolidation, and retrieval of memory processes in the PA test in mice (Figure 1A–C, appropriately).

In the next step, we assessed the impact of CBD or/and rivastigmine on the memory impairment provoked by an acute injection of scopolamine during these three stages of long-term memory. Based on the results obtained from the above-described pilot experiments, the non-effective doses of CBD (1 mg/kg) and rivastigmine (0.5 mg/kg) were then chosen for the next behavioural experiments evaluating the influence of these compounds administered alone or in combination on the memory impairment, provoked by an acute injection of scopolamine (1 mg/kg), using the PA test in mice.

### 2.2. The Influence of the Administration of CBD or/and Rivastigmine on the Memory Impairment Provoked by an Acute Administration of Scopolamine in the PA Test in Mice

#### Acquisition, Consolidation, and Retrieval of Memory

Two-way ANOVA analyses revealed that there was statistically significant effects:

- Acquisition of memory—caused by pretreatment [F(3.69) = 17.85; *p* < 0.0001], as well as a statistically significant effect caused by interactions [F(3.69) = 3.471; *p* = 0.0207], but there are no statistically significant effects caused by treatment [F(1.69) = 0.007856; *p* = 0.9296].
- Consolidation of memory—caused by pretreatment [F(3.64) = 19.82; *p* < 0.0001], as well as a statistically significant effect caused by interactions [F(3.64) = 2.924; *p* = 0.0405], but there are no statistically significant effects caused by treatment [F(1.64) = 0.1437; *p* = 0.7059].
- Retrieval of memory—caused by pretreatment [F(3.60) = 20.75; *p* < 0.0001], but there are no statistically significant effects caused by treatment [F(1.60) = 0.3279; *p* = 0.5690] and no statistically significant effects caused by interactions [F(3.60) = 1.656; *p* = 0.1860].

The post hoc Tukey’s test confirmed that scopolamine, at a dose of 1 mg/kg, significantly decreased LI values in mice in the PA test in comparison to the vehicle/vehicle/vehicle-treated mice, pointing to the amnestic effect of this drug:

- Acquisition of memory—scopolamine (*p* < 0.01);
- Consolidation of memory—scopolamine (*p* < 0.01);
- Retrieval of memory—scopolamine (*p* < 0.05).

What is of interest, for memory acquisition and memory consolidation, as well as for memory retrieval after an acute injection of CBD (1 mg/kg), is that an acute injection of rivastigmine (0.5 mg/kg) had no statistical influence on the memory-related processes in LI values in comparison to the vehicle/vehicle/vehicle-treated group.

However, the combined treatment of CBD (1 mg/kg) with rivastigmine (0.5 mg/kg) caused the statistical increase in LI values in comparison to the vehicle/vehicle/vehicle-treated group, indicating the positive influence of this combination on the memory consolidation processes (*p* < 0.05) or retrieval processes (*p* < 0.01), but not acquisition.

Moreover, the combined treatment of CBD (1 mg/kg) with rivastigmine (0.5 mg/kg) caused the statistical increase in LI values in comparison to the CBD (1 mg/kg)/vehicle/vehicle-treated group in acquisition of memory (*p* < 0.05) and consolidation of memory (*p* < 0.05), but not retrieval.

Moreover, an acute injection of a single dose of CBD (1 mg/kg) or rivastigmine (0.5 mg/kg) significantly attenuated changes in scopolamine-induced memory disturbances for both drugs, in comparison to the vehicle/vehicle/scopolamine (1 mg/kg)-treated group):

- Acquisition of memory—(*p* < 0.001 for vehicle/rivastigmine (0.5 mg/kg)/scopolamine (1 mg/kg) group and for CBD (1 mg/kg)/vehicle/scopolamine (1 mg/kg) group);
- Consolidation of memory (*p* < 0.001 for vehicle/rivastigmine (0.5 mg/kg)/scopolamine (1 mg/kg) group and *p* < 0.05 for CBD (1 mg/kg)/vehicle/scopolamine (1 mg/kg) group);
- Retrieval of memory—(*p* < 0.001 for vehicle/rivastigmine (0.5 mg/kg)/scopolamine (1 mg/kg) group and *p* < 0.05 for CBD (1 mg/kg)/vehicle/scopolamine (1 mg/kg) group).

**Figure 2:**
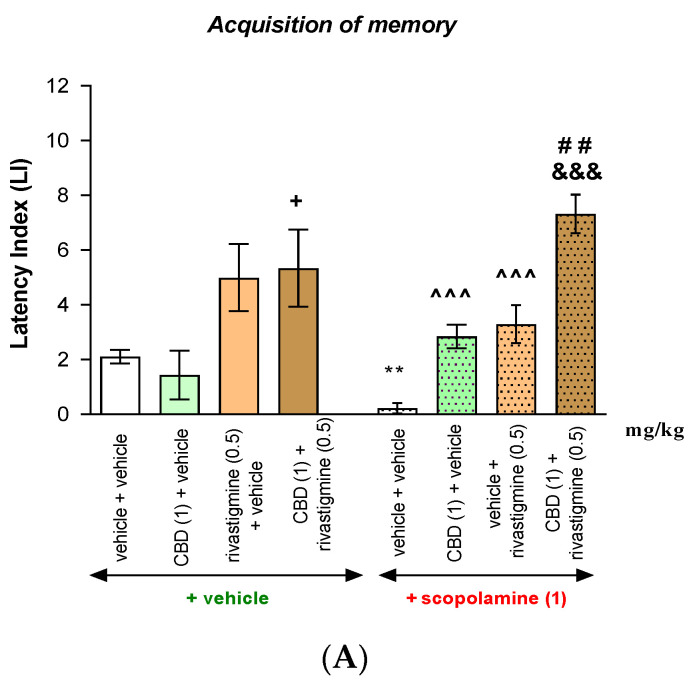

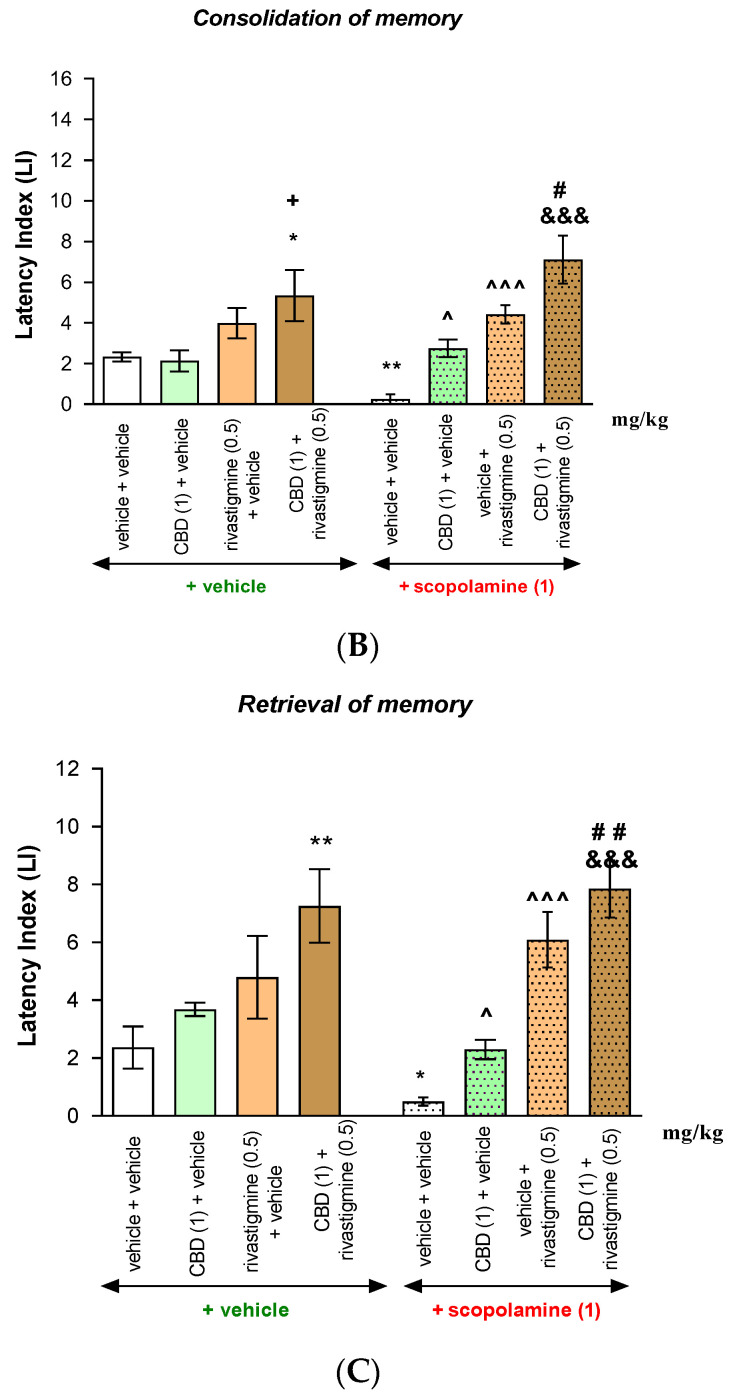
Influence of an acute administration of cannabinoid compounds—cannabidiol (CBD) (1 mg/kg) or/and AChEI—rivastigmine (0.5 mg/kg) on the memory impairment provoked by an acute injection of cholinergic receptor antagonist—scopolamine (1 mg/kg) expressed as a latency index (LI) during the acquisition (**A**), consolidation (**B**), and retrieval (**C**) memory using the PA test in mice. Non-effective dose of CBD (1 mg/kg) or/and rivastigmine (0.5 mg/kg) or/and vehicle were administered at 15 min intervals prior to amnestic dose of scopolamine (1 mg/kg). Injections of scopolamine were performed 15 min before the first trial (acquisition of memory), immediately after the first trial (consolidation of memory), or immediately before the second trial (retrieval of memory). The second trial was conducted 24 h later; the means ± SEM; * *p* < 0.05; ** *p* < 0.01 vs. vehicle/vehicle/vehicle group; ^ *p* < 0.05; ^^^ *p* < 0.001 vs. vehicle/vehicle/scopolamine (1 mg/kg)-treated group; + *p* < 0.05 vs. CBD (1 mg/kg)/vehicle/vehicle group; # *p* < 0.05; ## *p* < 0.01 vs. vehicle/rivastigmine (0.5 mg/kg)/scopolamine (1 mg/kg)-treated group; &&& *p* < 0.001 vs. CBD (1 mg/kg)/vehicle/scopolamine (1 mg/kg)-treated group; Tukey’s test; n = 8–12 (the exact number of mice in a test are presented in the table below (Table 2A–C)).

What is of interest is that co-administration of the non-effective, in the PA test, dose of CBD (1 mg/kg) and rivastigmine (0.5 mg/kg) attenuated memory impairment provoked by scopolamine (1 mg/kg) injection in the PA test in mice more significantly than a single administration of CBD (1 mg/kg) or single administration of rivastigmine (0.5 mg/kg):

- Acquisition of memory—(*p* < 0.001 in comparison to the CBD (1 mg/kg)/vehicle/scopolamine (1 mg/kg)-treated group and *p* < 0.01 in comparison to the vehicle/rivastigmine (0.5 mg/kg)/scopolamine (1 mg/kg)-treated group);
- Consolidation of memory—(*p* < 0.001 in comparison to the CBD (1 mg/kg)/vehicle/scopolamine (1 mg/kg)-treated group and *p* < 0.05 in comparison to the vehicle/rivastigmine (0.5 mg/kg)/scopolamine (1 mg/kg)-treated group);
- Retrieval of memory—(*p* < 0.001 in comparison to the CBD (1 mg/kg)/vehicle/scopolamine (1 mg/kg)-treated group and *p* < 0.01 in comparison to the vehicle/rivastigmine (0.5 mg/kg)/scopolamine (1 mg/kg)-treated group (Figure 2A–C, appropriately)).

## 3. Discussion

Confronting the theories presented in Section 1 concerning the pathology and/or treatment of AD and the information containing the complex beneficial role of ECS against memory and learning disorders typical for many neurodegenerative diseases, e.g., AD, the aim of the present study was to investigate the effects of a single administration of cannabinoid compound, CBD and AChEI, rivastigmine on the different stages of engram formation in long-term memory in mice in the PA test. Following that, we were going to determine and compare for the first time the effect of co-administration of CBD and rivastigmine on the memory disorders connected with cholinergic dysfunctions in experimental animal models. The assessment of cognitive processes in experimental animals was performed using the PA test, which is discussed in detail in Section 4.

Disturbances in the cholinergic system are an essential part of clinical symptoms of AD and always leads to abnormalities in memory functioning. Therefore, memory loss was induced in our experiments with the reference substance scopolamine, which belongs to the group of natural cholinolytic agents [15]. Scopolamine is a non-selective M-receptor antagonist and by blocking these receptors, this drug reduces the efficacy of ACh. By preventing ACh from attaching to the M receptor, it induces memory deficits. Given its mechanism of action, scopolamine is very often used as a pharmacological model of cognitive impairment accompanying diseases in which cholinergic pathways are involved, such as AD. The results of the study presented in the following paper using the PA test confirmed that, in mice, a single injection of scopolamine (0.3 and 1 mg/kg but not 0.1 mg/kg) negatively affects cognitive processes in all three phases (acquisition, consolidation, and retrieval) compared to a saline-treated control group. Based on these results, in our next experiments, scopolamine was used as a pharmacology model of impairment memory and learning at a dose of 1 mg/kg. Our results are consistent with data reported in the literature. According to the study, healthy young adults who were administered scopolamine experience memory impairments similar to those observed in patients with dementia [16].

As we mentioned, memory loss and cognitive deficits occurring in AD is due to a deficiency of ACh as a result of selective degenerative/death of cholinergic neurons. Current therapies of AD are aimed mainly at improving cholinergic transmission with the CNS. Rivastigmine is a reversible AChEI, used in an early pathophysiological feature of AD. This drug enhances cholinergic function, by increasing the concentration of ACh through reversible inhibition of its hydrolysis by cholinesterase. Given this mechanism, it can be inferred that the therapeutic effect of rivastigmine decreases with disease progression, as the number of damaged cholinergic neurons increases. Rivastigmine is used to treat mild-to-moderate AD-induced dementia. It has been shown that rivastigmine can treat cognitive impairment by inhibiting AChE and exert neuroprotective effects by affecting brain Aβ levels [17]. Our results with the PA test confirmed also that, in mice, a single injection of rivastigmine at doses of 1 and 2.5 mg/kg (but not 0.5 mg/kg) has a positive influence on the memory processes in all three phases (acquisition, consolidation, and retrieval) compared to a vehicle-treated control group in the PA test. We also revealed that an acute administration of the non-effective dose of rivastigmine (0.5 mg/kg) significantly affects changes in scopolamine-induced disturbances in three different memory stages (acquisition, consolidation, and retrieval) in mice in the PA test. Our results are in accordance with other experiments. These procognitive effects of rivastigmine and its ability to reverse scopolamine-induced memory impairment was confirmed by many preclinical studies using other animal memory tests, i.e., Morris water maze test (MWM) or radial arm maze test (RAM). Rivastigmine improved learning and enhanced memory in the rats with impaired scopolamine-induced memory. However, the efficacy of rivastigmine was observed to be limited, due to the narrow range of effective doses. Results showed that only at a dose of 0.5–2 mg/kg did it effectively reverse cognitive impairment. The efficiency of improvement in memory impairment was also dependent on the dose of scopolamine that was administered to the rodents [18]. Next, further clinical experiments tested the efficacy of rivastigmine in the treatment of cognitive impairment in AD. Currently, rivastigmine is one of the effective drugs used to reverse cognitive deficits in AD. However, this drug is not quite an effective therapy in AD [19,20]. Additionally, despite such beneficial procognitive effects of rivastigmine, it should be noted that numerous side effects occur with high frequency when rivastigmine is used during AD pharmacotherapy, especially when administered orally. The most common side effects of rivastigmine are gastrointestinal disorders, i.e., nausea, vomiting, diarrhoea, weight loss, and cardiovascular problems such as bradycardia. In addition, neuropsychiatric disorders including disorientation, sleep disturbances, dizziness, anxiety, and contact dermatitis may occur with transdermal patches [17,21]. Therefore, new treatment strategies for cognitive disorders are being sought, either as monotherapy or as an alternative method of pharmacotherapy.

One of the possible strategies for the modulation of memory and learning-related problems is connected with the influence on the ECS function. There is preclinical and clinical evidence for the potential of CBD to regulate different memory types [22,23,24,25,26,27]. For example, chronic use of CBD has been shown to improve working memory in the T-maze test and to increase the preference for a novel object in the novel object recognition (NOR) test in mice. Our results presented in this manuscript confirmed the procognitive influence of CBD, indicating that a single high-dose CBD injection (30 mg/kg) significantly improves memory in all three phases (acquisition, consolidation, and retrieval) compared to a vehicle-treated control group in mice using the PA test. However, it should be noted that there is research describing an opposite influence of CBD on cognition, i.e., an acute administration of CBD affects the so-called fear memory. One such experience describes how a systemic acute CBD administration before fear conditioning resulted in attenuated fear expression during later memory retrieval testing, indicating that the acute administration of CBD compromised the acquisition of fear learning [28].

The previously mentioned, well-documented antioxidant and anti-inflammatory effects and neuroprotective properties of CBD prompted researchers to test its effects in models of neurodegenerative disorders [29,30,31,32]. There are many literature data available in which researchers evaluate the positive influence of CBD on the prevention of the development of cognitive deficits in transgenic mice as models of AD (mice APPSwe/PS1E9 (APP × PS1)) [10,22,33,34,35,36,37,38,39,40,41]. Another experiment by Fagherazzi et al. [42] used an animal model of cognitive impairment induced by iron overload to test the effects of CBD (at a dose of 5.0 or 10.0 mg/kg) in rats with memory impairment. Iron-induced memory deficits are associated, among other things, with increased markers of oxidative stress in the brain. Thus, iron-induced cognitive impairment may be linked to oxidative damage, and the antioxidant properties of CBD may be beneficial in this context. Adult rats were administered CBD to observe the effects on memory impairment in three phases induced by iron administration in infancy assessed in the object recognition test (ORT) and PA. CBD was shown to be the best procognitive drug during the memory consolidation phase, while not enough of the desired effects were seen during the acquisition and retrieval phases [42,43].

These last cited experiments are particularly important in the context of the experiments and results presented in the work below. The results of our study showed that in mice, a single injection of the ineffective dose (1 mg/kg) of CBD reverses/alleviates the cognitive impairment induced by scopolamine (1 mg/kg) administration in mice in all memory phases assessed by the PA test. However, in the scientific literature, we can find effects that are opposite to ours. One study of Fadda et al. [44,45] assumed that cannabinoids can modulate multiple neurotransmitter systems, including glutamatergic and cholinergic, and attempted to prove that a CBD extract would be capable of reversing memory impairments induced by antagonists of these systems. Therefore, scopolamine, which is an M-receptor antagonist and causes memory deficits by blocking cholinergic transmission, was administered to some individuals. For the second group of rodents, MK-801, which is an NMDA receptor antagonist that blocks glutamatergic transmission, was administered. Two doses of CBD-rich extracts (5 and 10 mg/kg), which did not affect working memory when given alone, were unable to reverse these deficits when co-administered with scopolamine or MK801 in rats in an MWM test [44,45].

The differences in the results may be due to the fact that Fadda’s study used a CBD extract that contained other cannabinoids in small amounts, which may have interfered with the effects of CBD, while in our experiment, we used pure CBD. Furthermore, as previously mentioned, the therapeutic effect of CBD is dose-dependent; hence, differences in concentration may have influenced the differences in results, as well as different experimental procedure. However, most results, as well as ours, indicate a positive impact of CBD on memory and learning.

The neuroprotective and precognitive effects of CBD described above are connected with multiple mechanism of action of this compound. CBD has a low affinity for CB1 and CB2 receptors, but this compound indirectly activates CB1 via inhibition of FAAH, which aids in the accumulation of AEA. Thus, CBD could prevent glutamate-provoked excitotoxicity on neurons [46]. It is worth mentioning that CBD is also an inverse agonist of the CB2 receptor and thereby can inhibit microglial activation in a CB2 receptor-connected pathway [47]. Moreover, high anti-inflammatory and antioxidant activities after CBD are mediated via multiple molecular targets other than CB1 and CB2 receptors, in the CB receptor-independent pathway. CBD can prevent glutamate-induced neurotoxicity occurring by NMDA receptors, kainite receptor or 2-amino-3-hydroxy-5methyl-4-isoxazolepropionic acid (AMPA), peroxisome proliferation-activated receptor gamma (PPARγ), or adenosine receptors (A2A) [39,48]. CBD can also act by serotonin receptors (5-HT1A) or transient receptor potential cation channel subfamily V member 1 (TRPV1) [49,50].

Naturally, neither CBD nor rivastigmine provide lasting or perfect effects. It is known that compounds administered individually are often less effective. Polytherapy frequently provides longer-lasting effects, thus limiting the doses and side effects of drugs used separately. For that reason, it is necessary to search for an alternative method of pharmacotherapy in the context of AD. Therefore, new/old modified therapeutic options are being sought that would give more favourable treatment effects in the pharmacotherapy of AD. However, the effects of CBD and other procognitive drugs on memory disorders have not yet been elucidated.

Following that, in this study, we compared for the first time the effects of a single administration of CBD or rivastigmine and a single co-administration of these two drugs on the different stages of long-term memory trace formation in mice and on memory impairment induced by scopolamine administration in the PA test. We revealed that an acute co-administration of CBD (1 mg/kg) and rivastigmine (0.5 mg/kg) in ineffective doses positively affected cognitive processes and attenuated memory impairment provoked by scopolamine (1 mg/kg) injection in the PA test in mice, but at too much of a greater extent than when administered alone. This study using the combined administration of CBD and rivastigmine is, at this point in time, the only such experiment conducted. It is a novel study and therefore the results obtained cannot be compared with other data. There are little literature data describing the close relationship between the compounds modulating the functioning of the ECS, e.g., CBD and scopolamine. In the experiments presented in this study, interesting conclusions regarding this relationship were observed. The modulating effect of the compounds affecting the functioning of the ECS on the amnestic effects induced by scopolamine confirm the previously described relationship between the ECS and the cholinergic system and is consistent with the available literature data [11,51,52,53].

In the context of our results of co-administration of CBD and rivastigmine, the last available literature date seems to be interesting. To further enhance the therapeutic efficacy of CBD, it was chemically conjugated to a fragment of an AChE inhibitor. The new CBD-carbamate hybrid lead compound (C16) was able to improve scopolamine-induced cognition impairment in mice in MWM test and the treated mice exhibited better behavioural activity than donepezil [54].

The elaborated results of such a combination presented in the following paper represent preliminary research and perhaps offer an opportunity to create new perspectives in the treatment of cognitive disorders, including AD. However, further studies should be conducted at the clinical trial level to assess the effects of such polytherapy in more detail. As is well known, rivastigmine improves cognitive function in AD, while CBD shows a number of beneficial properties that may be relevant in the treatment of AD [29,30,31,32]. Linking all of these interventions through combination therapy offers great hope for advances in AD treatment. There is a possibility that such treatment will not only be effective in controlling symptoms but may lead to halting the progression of neurodegenerative diseases. The use of drugs that act not only on the cholinergic or glutamatergic system, but also on the ECS, expands the possibilities of treating cognitive disorders more effectively. On the other hand, polytherapy with rivastigmine and CBD may be as or more effective than monotherapy with the currently used rivastigmine, but the combined administration of these two compounds offers the possibility of reducing doses while maintaining the desired pharmacological effect. Thus, the risk of possible side effects after rivastigmine monotherapy is reduced.

Taking into account the above literature data and the results obtained during the study using the PA test, it can be concluded that one of the main phytocannabinoids, CBD, supports the formation of memory traces at all stages of memory: acquisition, consolidation, and retrieval. In addition, experience with the use of a combination of CBD and rivastigmine clearly shows a positive effect on alleviating cognitive disorders associated with cholinergic system dysfunction.

## 4. Materials and Methods

### 4.1. Animals

The experiments were carried out on naive male Swiss mice weighing 20–30 g. The animals were maintained under standard laboratory conditions (12 h light/dark cycle, room temperature at 21 ± 1 °C) with free access to tap water and laboratory feeding in their home cages and were adapted to the laboratory conditions for at least 1 week. Each experimental group consisted of 8–10 animals. All experiments were carried out according to the permission of the Local Ethical Committee (Local Ethical Committee for Animal Experiments in Lublin: Approval Code: 13/2020; Approval Date: 24 February 2020).

All behavioural experiments were conducted according to the National Institute of Health Guidelines for the Care and Use of Laboratory Animals and to the European Community Council Directive for the Care and Use of laboratory animals of 22 September 2010 (2010/63/EU). The authors complied with the ARRIVE guidelines to improve the reporting of animal research and the quality of the studies.

### 4.2. The Compounds Which Were Tested

- CBD (1 mg/kg) (Tocris Bioscience a Bio-Techne Brand, Biotechne, Warsaw, Poland)—a CB receptor ligand;
- Rivastigmine (0.5 mg/kg) (Tocris Bioscience a Bio-Techne Brand, Biotechne, Warsaw, Poland)—an AChEI;
- Scopolamine (1 mg/kg) (Tocris Bioscience a Bio-Techne Brand, Biotechne, Warsaw, Poland)—a cholinergic muscarinic receptor antagonist.

All compounds were suspended in a 1% solution of Tween 80 (Sigma, St. Louis, MO, USA) in saline solution (0.9% NaCl) and administered intraperitoneally (ip) at a volume of 10 mL/kg. Fresh drug solutions were prepared on each day of experimentation. Control groups received injections of saline with Tween 80 (vehicle) at the same volume and by the same route of administration.

### 4.3. Experimental Procedure

To assess and understand the memory-related effects, we used the passive avoidance (PA) test (obtained from ATANER, Lublin, Poland) commonly used to examine different stages of memory.

The apparatus of the PA consisted of a two-compartment acrylic box with a lighted compartment and darkened compartment. The light chamber was illuminated by a fluorescent light (8 W) and was connected to the dark chamber, which was equipped with an electric grid floor. Entrance of animals to the dark box was punished by an electric foot shock (0.2 mA for 2 s).

On the first day of training (pretest), mice were placed individually into the light compartment and were allowed to explore the light box (habitation). After 30 s, the guillotine door was raised to allow the mice to enter the dark compartment. When the mice entered the dark compartment, the guillotine door was closed and an electric foot-shock (0.2 mA) of 2 s duration was delivered immediately to the animal via grid floor. The latency time for entering the dark compartment was recorded (TL1).

**Figure 3:**
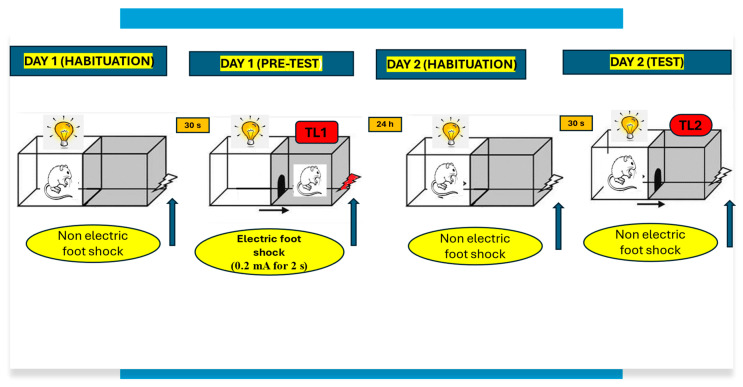
Procedure of the passive avoidance (PA) test.

Then, 24 h later, in the subsequent trial (retention, test), the same mice were again placed individually in the light compartment of the PA apparatus. After a 30 s adaptation period in the light (safe) chamber (habituation), the door between the compartments was raised and the time taken to re-enter the dark compartment was recorded (TL2) (Figure 3).

For the memory-related responses, the changes in PA performance were expressed as the difference between retention and training latencies and were taken as a latency index (LI). An LI was calculated for each animal and was reported as the ratio:

LI = TL2 − TL1/TL1

where:

TL1—the time taken to enter the dark compartment during the training;
TL2—the time taken to re-enter the dark compartment during the retention [55].

Depending on the procedure used, the PA test allows examining different durations of memory (short-term and long-term memory) according to the period between training and test, as well as different stages of memory (acquisition, consolidation, and retrieval) according to the time of drug treatment. When mice were tested 24 h after TL1, long-term fear memory was assessed. Drug administration before the first trial (before pretest) should interfere with the acquisition of information and drug administration immediately after the first trial (after pretest) should exert an effect on the process of consolidation, while the administration of tested compounds before the second trial (before test) should interfere with the retrieval of memory information [55].

The experimental doses of drugs used for behavioural experiments and procedures were chosen accordingly to those frequently used in the literature and our previous experiences [11,25,26,55,56].

### 4.4. Treatment

**Figure 4:**
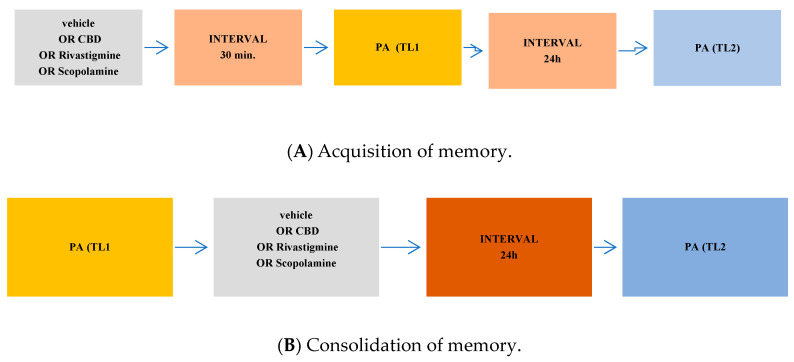

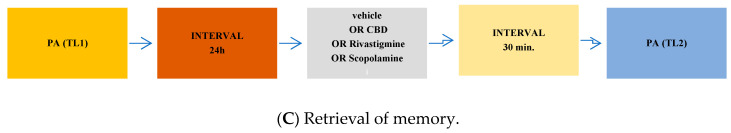
The scheme of a single drug administration and behavioural experiments during the assessment of memory acquisition (**A**), consolidation (**B**), or retrieval (**C**) in the PA test in mice.

The first step of the experiment was designed to estimate the influence of selected compounds: phytocannabinoid compounds—CBD or AChEI—rivastigmine or cholinergic muscarinic receptor antagonist—scopolamine on the different stages (acquisition, consolidation, and retrieval) of long-term memory in mice, using the PA test. CBD (1, 5, 30 mg/kg; ip), rivastigmine (0.5, 1, 2.5 mg/kg; ip), scopolamine (0.1, 0.3, 1 mg/kg; ip), or vehicle, for the control group, were administered 30 min before the first trial (acquisition of memory) (Figure 4A), immediately after the first trial (consolidation of memory) (Figure 4B), or before the second trial (retrieval of memory). The second trial was conducted 24 h later and retested after 24 h (long-term memory) (Figure 4C).

**Figure 5:**
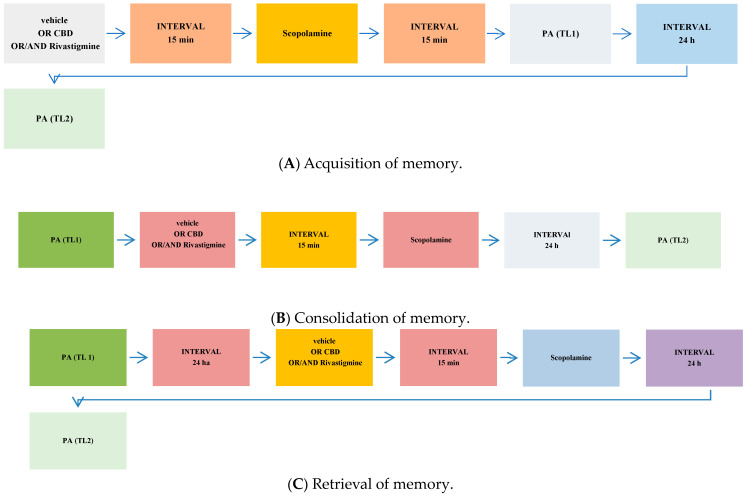
The scheme of co-administration of drugs and behavioural experiments during the assessment of memory acquisition (**A**), consolidation (**B**), or retrieval (**C**) in the PA test in mice.

Next, based on these pilot experiments, we chose the non-effective doses of CBD and rivastigmine for the subsequent experiment with an effective dose of scopolamine. We assessed the impact of CBD or/and rivastigmine on the memory impairment provoked by an acute injection of scopolamine during the three stages of long-term memory, using the PA test in mice. The non-effective dose of CBD (1 mg/kg) or/and rivastigmine (0.5 mg/kg) or/and vehicle were administered at 15 min intervals, 15 min prior to the amnestic dose of scopolamine (1 mg/kg). Injections of scopolamine were performed 15 min before the first trial (acquisition of memory) (Figure 5A), immediately after the first trial (consolidation of memory) (Figure 5B), or immediately before the second trial (retrieval of memory) (Figure 5C). The second trial was conducted 24 h later.

### 4.5. Statistical Analysis

The statistical analysis was performed using one-way or two-way analysis of variance (ANOVA) for the factors of pretreatment, treatment, and pretreatment/treatment interactions. Post hoc comparison of means was carried out with Tukey’s test (for one-way and two-way ANOVA) for multiple comparisons, when appropriate.

The data were considered statistically significant at a confidence limit of *p* < 0.05. ANOVA analysis with the post hoc Tukey’s test was performed using GraphPad Prism version 7 for Windows (GraphPad Software, SanDiego, CA, USA, www.graphpad.com, accessed on 5 July 2024).

For the memory-related behaviours, the changes in PA performance were expressed as the difference between retention and training latencies and were taken as an LI calculated for each animal and were reported as the ratio mentioned before [55].

## 5. Conclusions

- Single, acute administration of CBD (1 mg/kg; ip) significantly affected changes in scopolamine-induced disturbances in memory acquisition and consolidation.
- Single, acute administration of rivastigmine (0.5 mg/kg; ip) significantly affected changes in scopolamine-induced disturbances in memory acquisition.
- Co-administration of non-effective, in the PA test, doses of CBD (1 mg/kg; ip) and rivastigmine (0.5 mg/kg; ip) attenuated, more significantly than single administration of the used drugs, memory impairment provoked by scopolamine (1 mg/kg; ip) injection in the PA test in mice.

As such, the combination therapy of these two compounds appears to be more beneficial in reducing scopolamine-induced cognitive impairment than substances administered alone. This combination of two drugs, CBD and rivastigmine, seems to be favourable in the pharmacotherapy of cognitive disorders, connecting with cholinergic pathways. The expanded knowledge of this topic allows us to plan further research on new, more effective pharmacological strategies for the treatment of memory impairment, which is one of the predominant symptoms of AD. This kind of polytherapy could increase whole therapeutic effectiveness.

## Figures and Tables

**Table 1:** The number of animals used in each group for each parameter; (VEH—vehicle; CBD—cannabidiol; RIV—rivastigmine; SCOP—scopolamine); for Acquisition of memory (**A**); Consolidation of memory (**B**); Retrieval of memory (**C**).

**(A)**										
	**VEH**	**CBD 1**	**CBD 5**	**CBD 30**	**RIV 0.5**	**RIV 1**	**RIV 2.5**	**SCOP 0.1**	**SCOP 0.3**	**SCOP 1**
**Number of Mice**	**11**	**12**	**7**	**8**	**8**	**8**	**8**	**8**	**8**	**10**
Mininmum	0.98	−0.69	1.04	1.57	0.41	4.39	4.95	0.95	0.95	−0.11
Maximum	3.27	8.64	2.52	7.60	12.79	15.95	22.96	2.26	1.97	0.95
Range	2.29	9.33	1.47	6.03	12.38	11.55	18.01	1.31	1.02	1.06
Mean	2.20	1.40	1.67	5.07	4.54	11.03	15.40	1.78	1.29	0.31
Std. Deviation	0.81	2.66	0.60	2.06	4.45	3.65	6.58	0.56	0.43	0.46
Std. Error of Mean	0.24	0.77	0.23	0.73	1.57	1.29	2.33	0.20	0.15	0.14
**(B)**										
	**VEH**	**CBD 1**	**CBD 5**	**CBD 30**	**RIV 0.5**	**RIV 1**	**RIV 2.5**	**SCOP 0.1**	**SCOP 0.3**	**SCOP 1**
**Number of Mice**	**7**	**9**	**7**	**7**	**10**	**8**	**7**	**8**	**8**	**12**
Mininmum	0.07	−0.59	0.67	1.50	−0.80	5.00	4.83	0.05	−0.36	−0.77
Maximum	3.69	2.67	6.47	11.30	12.64	20.43	32.33	2.47	2.05	1.63
Range	3.62	3.26	5.81	9.80	13.44	15.43	27.50	2.42	2.41	2.40
Mean	3.15	1.30	2.99	6.67	4.09	13.66	17.91	1.36	0.96	0.49
Std. Deviation	1.40	0.91	2.51	3.93	4.34	5.85	10.44	0.95	0.89	0.81
Std. Error of Mean	0.53	0.30	1.12	1.49	1.37	2.07	3.95	0.34	0.32	0.23
**(C)**										
	**VEH**	**CBD 1**	**CBD 5**	**CBD 30**	**RIV 0.5**	**RIV 1**	**RIV 2.5**	**SCOP 0.1**	**SCOP 0.3**	**SCOP 1**
**Number of Mice**	**8**	**8**	**8**	**8**	**9**	**10**	**8**	**8**	**8**	**8**
Mininmum	1.52	2.78	2.13	4.32	1.41	1.31	5.25	0.81	0.39	−0.66
Maximum	3.72	6.00	7.16	7.46	15.29	14.79	25.27	3.11	2.15	0.95
Range	2.20	3.22	5.02	3.14	13.87	13.48	20.02	2.30	1.76	1.61
Mean	2.42	3.92	4.16	6.19	5.96	9.43	16.78	1.65	1.28	0.49
Std. Deviation	0.65	1.15	1.74	1.20	5.16	4.70	7.75	0.85	0.70	0.50
Std. Error of Mean	0.23	0.41	0.61	0.43	1.72	1.49	2.74	0.30	0.25	0.18

**Table 2:** The number of animals used in each group for each parameter; (VEH—vehicle; CBD—cannabidiol; RIV—rivastigmine; SCOP—scopolamine); for Acquisition of memory (**A**); Consolidation of memory (**B**); Retrieval of memory (**C**).

**(A)**								
	**VEH/VEH/VEH**	**CBD/VEH/VEH**	**RIV/VEH/VEH**	**CBD/RIV/VEH**	**VEH/VEH/SCOP**	**CBD/VEH/SCOP**	**VEH/RIV/SCOP**	**CBD/RIV/SCOP**
**Number of Mice**	**10**	**10**	**9**	**8**	**12**	**10**	**8**	**10**
Mininmum	0.98	−0.53	1.41	1.43	−0.95	1.29	0.45	4
Maximum	3.27	8.64	12.64	10.06	1.35	4.83	6.46	10.63
Range	2.29	9.16	11.22	8.63	2.29	3.55	6.00	6.63
Mean	2.10	1.43	4.99	5.34	0.22	2.85	3.29	7.32
Std. Deviation	0.77	2.80	3.68	3.97	0.68	1.37	1.95	2.25
Std. Error of Mean	0.24	0.89	1.23	1.40	0.20	0.43	0.69	0.71
**(B)**								
	**VEH/VEH/VEH**	**CBD/VEH/VEH**	**RIV/VEH/VEH**	**CBD/RIV/VEH**	**VEH/VEH/SCOP**	**CBD/VEH/SCOP**	**VEH/RIV/SCOP**	**CBD/RIV/SCOP**
**Number of Mice**	**10**	**8**	**9**	**8**	**10**	**9**	**9**	**9**
Mininmum	1.04	0.43	2.04	1.43	−0.95	0.55	2.92	1.13
Maximum	3.27	4.52	9.00	10.06	0.95	4.57	6.47	11.42
Range	2.23	4.09	6.96	8.63	1.91	4.03	3.54	10.28
Mean	2.33	2.13	3.99	5.34	0.25	2.75	4.42	7.11
Std. Deviation	0.73	1.46	2.25	3.57	0.74	1.27	1.35	3.54
Std. Error of Mean	0.23	0.52	0.75	1.26	0.23	0.42	0.45	1.18
**(C)**								
	**VEH/VEH/VEH**	**CBD/VEH/VEH**	**RIV/VEH/VEH**	**CBD/RIV/VEH**	**VEH/VEH/SCOP**	**CBD/VEH/SCOP**	**VEH/RIV/SCOP**	**CBD/RIV/SCOP**
**Number of Mice**	**9**	**9**	**8**	**8**	**10**	**8**	**8**	**8**
Mininmum	−0.47	3.26	1.41	3.08	−0.66	0.69	1.52	3.00
Maximum	5.71	5.38	12.64	11.20	0.95	3.17	8.93	11.00
Range	6.19	2.12	11.22	8.12	1.61	2.48	7.41	8.00
Mean	2.37	3.68	4.79	7.26	0.49	2.30	6.09	7.85
Std. Deviation	2.18	0.69	4.05	3.58	0.45	0.94	2.72	2.80
Std. Error of Mean	0.73	0.23	1.43	1.27	0.14	0.33	0.96	0.99

## Data Availability

Data is contained within the article.
